# Differences in Diabetic Prescription Drug Utilization and Costs Among Patients With Diabetes Enrolled in Colorado Marketplace and Medicaid Plans, 2014-2015

**DOI:** 10.1001/jamanetworkopen.2021.40371

**Published:** 2022-01-14

**Authors:** Peggah Khorrami, Michael S. Sinha, Aditi Bhanja, Heidi L. Allen, Aaron S. Kesselheim, Benjamin D. Sommers

**Affiliations:** 1 Department of Health Policy and Management, Harvard T. H. Chan School of Public Health, Boston, Massachusetts; 2 Harvard-MIT Center for Regulatory Science, Harvard Medical School, Boston, Massachusetts; 3 Program on Regulation, Therapeutics, and Law, Division of Pharmacoepidemiology and Pharmacoeconomics, Department of Medicine, Brigham & Women’s Hospital, Harvard Medical School, Boston, Massachusetts; 4 Columbia University School of Social Work, New York, New York; 5 Department of Medicine, Brigham and Women’s Hospital, Boston, Massachusetts

## Abstract

**Importance:**

Increasing prices of antidiabetic medications in the US have raised substantial concerns about the effects of drug affordability on diabetes care. There has been little rigorous evidence comparing the experiences of patients with diabetes across different types of insurance coverage.

**Objective:**

To compare the utilization patterns and costs of prescription drugs to treat diabetes among low-income adults with Medicaid vs those with Marketplace insurance in Colorado during 2014 and 2015.

**Design, Setting, and Participants:**

This cross-sectional study included diabetic patients enrolled in Colorado Medicaid and Marketplace plans who were aged 19 to 64 years and had incomes between 75% and 200% of the federal poverty level during 2014 and 2015. Data analysis was conducted from September 2020 to April 2021.

**Exposures:**

Health insurance through Colorado Medicaid or Colorado’s state-based Marketplace.

**Main Outcomes and Measures:**

Primary outcomes were drug utilization (prescription drug fills) and drug costs (total costs and out-of-pocket costs). The secondary outcome was months with an active prescription for noninsulin antidiabetic medications. An all payer claims database was combined with income data, and linear models were used to adjust for clinical and demographic confounders.

**Results:**

Of 22 788 diabetic patients included in the study, 20 245 were enrolled in Medicaid and 2543 in a Marketplace plan. Marketplace-eligible individuals were older (mean [SD] age, 52.12 [10.60] vs 47.70 [11.33] years), and Medicaid-eligible individuals were more likely to be female (12 429 [61.4%] vs 1413 [55.6%]). Medicaid-eligible patients were significantly more likely than Marketplace-eligible patients to fill prescriptions for dipeptidyl peptidase 4 inhibitors (adjusted difference, −3.7%; 95% CI, −5.3 to −2.1; *P* < .001) and sulfonylureas (adjusted difference, −6.6%; 95% CI, −8.9 to −4.3; *P* < .001). Overall rates of insulin use were similar in the 2 groups (adjusted difference, −2.3%; −5.1 to 0.5; *P* = .11). Out-of-pocket costs for noninsulin medications were 84.4% to 95.2% lower and total costs were 9.4% to 54.2% lower in Medicaid than in Marketplace plans. Out-of-pocket costs for insulin were 76.7% to 94.7% lower in Medicaid than in Marketplace plans, whereas differences in total insulin costs were mixed. The percentage of months of apparent active medication coverage was similar between the 2 groups for 4 of 5 drug classes examined, with Marketplace-eligible patients having a greater percentage of months than Medicaid-eligible patients for sulfonylureas (adjusted difference, 5.3%; 95% CI, 0.3%-10.4%; *P* = .04).

**Conclusions and Relevance:**

In this cross-sectional study, drug utilization across multiple drug classes was higher and drug costs were significantly lower for adults with diabetes enrolled in Medicaid than for those with subsidized Marketplace plans. Patients with Marketplace coverage had a similar percentage of months with an active prescription as patients with Medicaid coverage.

## Key Points

**Question:**

How did utilization patterns and costs of prescription drugs to treat diabetes compare between Medicaid and Marketplace health insurance plans in Colorado during 2014 and 2015?

**Findings:**

In this cross-sectional study of 22 788 diabetic adults with incomes between 75% and 200% of the federal poverty level, drug utilization across multiple drug classes was higher and drug costs were significantly lower for patients enrolled in Medicaid than for patients with subsidized Marketplace plans.

**Meaning:**

In this study, patients with diabetes had broader access to expensive prescription drugs in Medicaid than in private plans, and pharmaceutical costs for these treatments were lower for Medicaid enrollees than for Marketplace plan enrollees.

## Teaser

This cross-sectional study compares diabetic drug utilization and cost patterns between Colorado Medicaid and Marketplace health insurance plans during 2014 and 2015.

## Introduction

Diabetes is a major contributor to morbidity and mortality in the US; 1 in 10 people have been diagnosed with diabetes, and 1 in 3 people are at increased risk of progressing to diabetes.^1^ The estimated national cost of diabetes in 2017 was $327 billion, with $237 billion representing direct health care expenditures and $90 billion representing indirect costs related to factors such as lost productivity, unemployment from chronic disability, and premature mortality.^2^ As the incidence and prevalence of diabetes have increased over the past 2 decades, so too have the prices of antidiabetic medications in the US, raising substantial concerns about the effects of drug affordability on diabetes care.

Perhaps the most commonly discussed issue regarding the cost of diabetes treatment is increasing insulin prices, which remain high owing to limited market competition.^3^ Although insulin was initially discovered and donated for the public good about a century ago, the insulin formulations sold today continue to be protected by multiple patents, including on the delivery device.^4^ However, insulin is not the only treatment affected by increasing costs. A 2019 study^5^ found that the list price for diabetes medications and supplies increased by 58% from 2014 to 2019, a rate nearly double that of the increase in overall drug prices during that period. In addition, prices for noninsulin diabetes medications increased at a faster rate than prices for insulin products (76% vs 50%) during that period. In addition to drug list prices, insurance coverage rules, such as those regarding patient cost-sharing, also affect whether patients can access antidiabetic medications and supplies.

The Patient Protection and Affordable Care Act of 2010 (ACA) allowed states to expand Medicaid coverage to millions of previously uninsured individuals with low income while also giving access to millions more patients with low income to enroll in subsidized private Marketplace insurance plans.^6^ An estimated 1.9 million people with diabetes are enrolled in Medicaid and Marketplace insurance plans.^7^ However, little is known about how well Medicaid compares with the subsidized private Marketplace insurance plans in providing adequate coverage for enrollees and in restraining costs. For example, access to medications such as insulin and noninsulin diabetic medications, particularly brand-name drugs in newer therapeutic classes, may differ between Medicaid and Marketplace insurance programs. Spending on diabetic drugs is also a major factor in state and federal health care budgets given the outsized effects of diabetes care on total medical expenditures.^8^

We therefore sought to evaluate prescription drug utilization and spending among patients enrolled in Medicaid vs those enrolled in Marketplace insurance plans. The experiences of patients with chronic conditions in these 2 types of insurance plans are relevant to ongoing policy discussions about how best to expand coverage to uninsured populations, including whether to prioritize government-provided insurance such as Medicaid or a public option and/or to increase subsidies encouraging more people to purchase private Marketplace insurance.^9^ The quality and affordability of health care in these 2 types of insurance for people with chronic conditions such as diabetes is therefore an important clinical and policy question. Our study objective was to compare the utilization patterns and costs of prescription drugs to treat diabetes among low-income individuals with Medicaid vs those with Marketplace insurance.

## Methods

### Data

This cross-sectional study used data from the Colorado all payer claims database (APCD) merged with income data from the state’s Medicaid and Marketplace programs. The APCD contains insurance claims from nearly all residents in the state, including complete Medicaid fee-for-service and managed care claims, claims from the 21 largest commercial payers, and 85% to 90% of state Marketplace claims.^10^ Specifically, the Colorado APCD includes data on insurance enrollment; utilization and payments for outpatient, inpatient, and prescription drugs; and beneficiary demographic characteristics.^11^ For 2014 and 2015, we combined this data set with detailed income eligibility information provided by the state’s Medicaid agency and Marketplace. The study was approved by the institutional review board at the Harvard T. H. Chan School of Public Health, and because we used only secondary deidentified data, a waiver of informed consent was obtained. This study followed the Strengthening the Reporting of Observational Studies in Epidemiology (STROBE) reporting guideline.

### Sample

We assessed differences in prescription drug use for diabetes among individuals enrolled in Medicaid or Marketplace insurance plans, limiting the sample to adults aged 19 to 64 years with incomes between 75% and 200% of the federal poverty level (FPL). By focusing on this income range, our study aimed to examine underlying differences in utilization across subsidized insurance types. Although individuals with higher income (>200% of FPL) can also enroll in Marketplace plans, these individuals likely differ substantially across both measured and unmeasured factors from those included in the study sample. Individuals with incomes between 75% and 138% of FPL are eligible for Medicaid, and those with higher incomes can purchase subsidized private insurance through the state’s ACA Marketplace. We excluded individuals who qualified for Medicaid owing to disability or pregnancy-related eligibility because those 2 groups have different income eligibility criteria for Medicaid. We also excluded Marketplace enrollees who had incomes below 138% of FPL because this group consists primarily of individuals who are ineligible for Medicaid based on immigration status.

The sample was limited to individuals with diabetes, which consistent with prior research,^12^ we defined based on either an *International Classification of Diseases, Ninth Revision* or *International Statistical Classification of Diseases and Related Health Problems, Tenth Revision* diagnosis code for diabetes or at least 1 prescription for diabetic medication during the calendar year. We describe the sample construction in the eFigure in the Supplement.

### Statistical Analysis

Our primary outcomes were drug utilization (prescription drug fills) and drug costs (total costs and out-of-pocket costs). For utilization, we classified antidiabetic medications into 5 categories of noninsulin medications (dipeptidyl peptidase 4 [DPP-4] inhibitors, glucagon-like peptide 1 [GLP-1] agonists, sodium-glucose transport protein 2 [SGLT-2] inhibitors, sulfonylureas, and metformin) and 3 categories of insulin (basal, meal-time, and combination insulins).

For costs, we assessed both total and out-of-pocket costs for the year as well as unit costs. Unit costs were defined based on the monthly cost for noninsulin medications. For insulin medications, the dosage information in the database did not allow us to precisely estimate the duration of a prescription. Instead, we analyzed the mean cost per prescription claim.

Secondary outcomes focused on months with an active prescription (as a proxy for medication adherence) for noninsulin antidiabetic medications. We concentrated on noninsulin antidiabetic medications because it is generally easier to track prescription duration compared with insulins. For the 5 noninsulin antidiabetic drug groups, we reported the percentage of months during which patients were enrolled in Medicaid or Marketplace insurance and had an active prescription for that medication (ie, adherence was measured as months with an active prescription/months of coverage after receiving an active prescription), with the sample limited in each case only to individuals who received any prescription for that particular drug class during the year. This metric resembles the adherence measures used in prior studies.^13,14^

For each set of outcomes, we compared individuals eligible for Medicaid (income of 75%-138% of the FPL) vs those eligible for Marketplace coverage (income of 139%-200% of the FPL). People whose income changed during the course of the year were classified in the coverage category based on their initial income during the year, consistent with an intent-to-treat approach. We calculated unadjusted means and proportions for each outcome comparing these 2 income groups, and then we used linear regression models to estimate the net difference between Marketplace and Medicaid groups for each outcome. Multivariable models adjusted for age, sex, Elixhauser comorbidity index, income, and urban vs rural residence. *P* < .05 was considered statistically significant, and all *P* values were 2-sided. The analyses were conducted from September 2020 to April 2021 using Stata, version 14.0 (StataCorp LLC).

## Results

Table 1 lists the overall sample characteristics (N = 22 788) by age, sex, and residence area. A total of 20 245 patients with diabetes (88.8%) were eligible for Medicaid, and 2543 (11.2%) were eligible for Marketplace plans. The Medicaid-eligible group had a mean (SD) income of 106% (18.5) FPL, and the Marketplace-eligible group had a mean (SD) income of 168% (17.5) FPL (approximately $12 500 vs $19 800 annually for an individual or $21 300 vs $33 800 for a family of 3, as of 2015). The mean (SD) months of coverage were 10.74 (2.45) for Medicaid-eligible patients and 9.73 (2.98) for Marketplace-eligible patients. Marketplace-eligible individuals were slightly older (mean [SD], 52.12 [10.60] vs 47.70 [11.33] years), and Medicaid-eligible individuals were more likely to be female (12 429 [61.4%] vs 1413 [55.6%]).

**Table 1.** Characteristics of Patients With Diabetes by Insurance Type^a^

Characteristic	Patients	
	Medicaid eligible (n = 20 245)^b^	Marketplace eligible (n = 2543)^c^
Income, mean (SD), % FPL	106 (18.50)	168 (17.49)
Duration of coverage, mean (SD), mo	10.74 (2.45)	9.73 (2.98)^d^
Age, mean (SD), y	47.70 (11.33)	52.12 (10.60)^d^
Age, No. (%)		
19-25 y	704 (3.5)	49 (1.9)^d^
26-34 y	2362 (11.7)	185 (7.3)^d^
35-44 y	4449 (22.0)	307 (12.1)^d^
45-54 y	5858 (28.9)	654 (25.7)^d^
55-64 y	6872 (33.9)	1348 (53.0)^d^
Sex, No. (%)		
Female	12 429 (61.4)	1413 (55.6)^d^
Male	7814 (38.6)	1125 (44.2)^d^
Elixhauser comorbidity index, mean (SD)	0.71 (1.39)	0.99 (1.56)^d^
Rural area of residence, No. (%)	1475 (7.3)	178 (7.0)

Abbreviation: FPL, federal poverty level.

^a^ Data are from the Colorado All Payer Claims Database, linked to income data from Medicaid and Marketplace eligibility files.

^b^ Patients with income of 75% to 138% of the FPL were eligible for Medicaid.

^c^ Patients with income of 139% to 200% of the FPL were eligible for Marketplace insurance.

^d^ Statistically significant at *P* < .01.

### Patients Using Insulin and Noninsulin Antidiabetic Medications

Table 2 presents the number of patients using insulin and noninsulin antidiabetic medications by insurance type, the most frequently used insulin brands and noninsulin antidiabetic drug groups, and the difference between Medicaid and Marketplace rates for each drug group (expressed as Marketplace compared with Medicaid). A total of 14 317 patients (73.9%) filled at least 1 prescription for a noninsulin antidiabetic medication. Similar proportions of patients in the Medicaid and Marketplace groups used a generic medication (adjusted difference, 1.5%; 95% CI, −1.3 to 4.2; *P* = .30), but brand-name prescriptions were less common in the Marketplace group (adjusted difference, −4.7%; 95% CI, −6.6 to −2.7; *P* < .001). The most commonly used medication in both groups was metformin (91.3% [95% CI, 90.6-92.0] of all patients using noninsulin antidiabetic medications). In the adjusted model, compared with Marketplace-eligible patients, Medicaid-eligible patients were significantly more likely to fill prescriptions for DPP-4 inhibitors (adjusted difference, −1.3; 95% CI, −9.7 to 7.0; *P* = .76) and sulfonylureas (adjusted difference, 5.3; 95% CI, 0.3-10.4; *P* = .04), whereas there were no significant differences for GLP-1 agonist and SGLT-2 inhibitor use.

**Table 2.** Diabetic Adults Using Insulin and Noninsulin Antidiabetic Medications per Year by Drug and Insurance Type^a^

Drug	Patients, No (%)		*P* value	Adjusted difference, % (95% CI)^d^	*P* value
	Medicaid eligible (n = 19 386)^b^	Marketplace eligible (n = 2432)^c^			
**Noninsulin antidiabetic medications**					
All	14 317 (73.9)	1802 (74.1)	.80	0.8 (−1.8 to 3.5)	.54
Any generic prescription	13 848 (71.4)	1756 (72.2)	.43	1.5 (−1.3 to 4.2)	.30
Any brand prescription	2332 (12.0)	164 (6.7)	<.001	−4.7 (−6.6 to −2.7)	<.001
Top drug groups					
DPP-4 inhibitors	1608 (8.3)	93 (3.8)	<.001	−3.7 (−5.3 to −2.1)	<.001
GLP-1 agonists	640 (3.3)	59 (2.4)	.02	−1.0 (−2.1 to 0.1)	.07
SGLT-2 inhibitors	323 (1.7)	34 (1.4)	.33	−0.6 (−1.4 to 0.1)	.11
Sulfonylureas	3506 (18.1)	296 (12.2)	<.001	−6.6 (−8.9 to −4.3)	<.001
Metformin	13 062 (67.4)	1657 (68.1)	.45	2.1 (−0.7 to 5.0)	.14
**Insulin medications** ^e^					
All	5877 (30.3)	713 (29.3)	.31	−2.3 (−5.1 to 0.5)	.11
Basal or background					
Insulin detemir injection (Levemir)	3666 (18.9)	137 (5.6)	<.001	−12.9 (−15.2 to −10.6)	<.001
Insulin glargine (Lantus)	2103 (10.8)	255 (10.4)	.59	0.6 (−1.3 to 2.5)	.55
Insulin isophane (Humulin N)	580 (3.0)	225 (9.3)	<.001	5.0 (3.8 to 6.2)	<.001
Bolus or meal-time					
Insulin lispro (Humalog)	1676 (8.6)	205 (8.4)	.72	−0.3 (−2.0 to 1.5)	.77
Insulin aspart (Novolog)	1608 (8.3)	101 (4.2)	<.001	−5.2 (−6.9 to −3.6)	<.001
Insulin human regular (Humulin R)	293 (1.5)	99 (4.1)	<.001	2.1 (1.3 to 2.9)	<.001
Premixed or combination					
70% Human insulin isophane suspension and 30% human insulin injection (Humulin 70/30)	155 (0.8)	33 (1.4)	.005	0.1 (−0.4 to 0.7)	.67
70% Human insulin isophane suspension and 30% regular human insulin injection (Novolin 70/30)	174 (0.9)	13 (0.5)	.07	−0.5 (−1.1 to 0.01)	.09
70% Insulin aspart protamine suspension and 30% insulin aspart injection (Novolog 70/30)	117 (0.6)	4 (0.2)	.006	−0.5 (−0.9 to 0.0)	.05

Abbreviations: DPP-4, dipeptidyl peptidase 4; GLP-1, glucagon-like peptide 1; SGLT-2, sodium-glucose transport protein 2.

^a^ Data are from the Colorado All Payer Claims Database, linked to income data from Medicaid and Marketplace eligibility files.

^b^ Patients with income of 75% to 138% of the federal poverty level were eligible for Medicaid.

^c^ Patients with income of 139% to 200% of the federal poverty level were eligible for Marketplace insurance.

^d^ Model adjusted for age, sex, Elixhauser comorbidity index, income, and urban vs rural residence.

^e^ The top 3 most commonly prescribed insulin brands in each category are presented. Some patients received more than 1 type of insulin during the year; thus, column percentages may sum to more than 100%.

Table 2 also presents rates of insulin prescriptions for the top 3 types of insulin in each category. Overall rates of insulin use were similar in the 2 groups (adjusted difference, −2.3%; 95% CI, −5.1 to 0.5; *P* = .11). Basal insulins were the most frequently used type of insulin, with insulin detemir (Levemir) more common in the Medicaid group and insulin isophane (Humulin N) more common in the Marketplace group; similar proportions in the groups used insulin glargine (Lantus). Among meal-time insulins, Medicaid enrollees were significantly more likely to use insulin aspart (Novolog) and Marketplace enrollees more likely to use insulin human regular (Humulin R), with similar rates of use for insulin lispro (Humalog). Combination insulins were filled at a lower rate than the other 2 categories, with some differences between insurance types.

### Cost of Treatment

**Figure 1.**
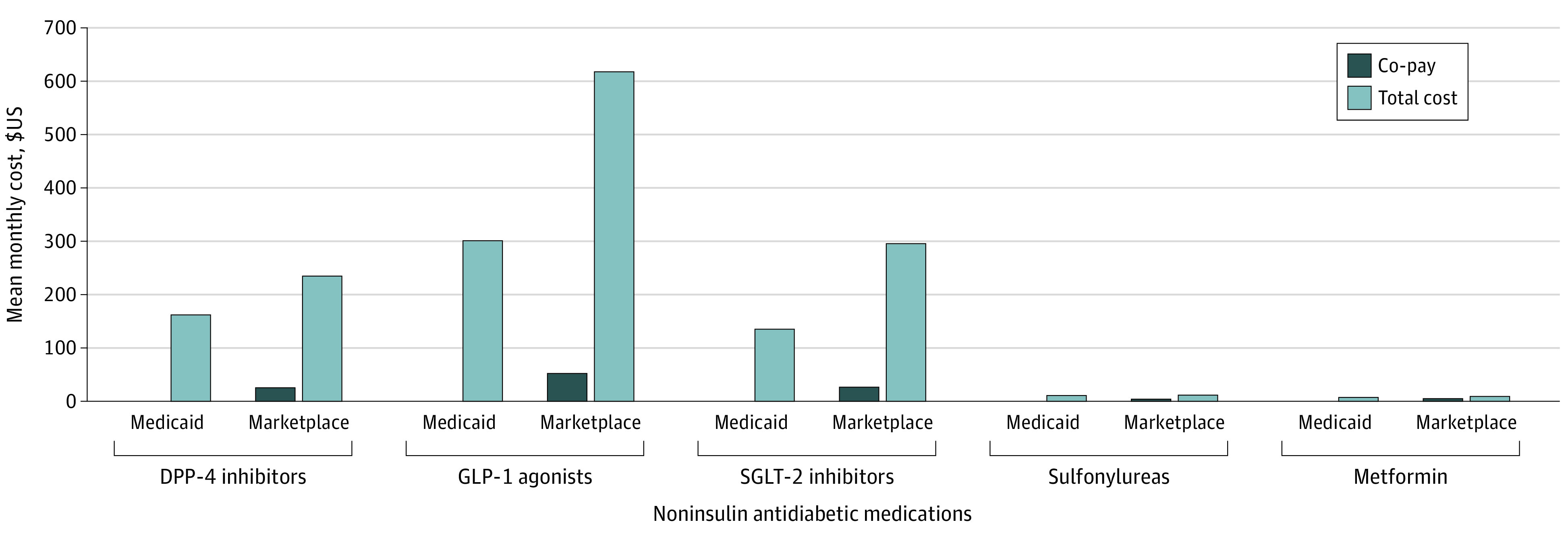
Monthly Cost of Noninsulin Antidiabetic Medications by Insurance Type

Figure 1 presents the mean co-pay per month and the mean total cost per month of noninsulin antidiabetic medications by insurance type. Out-of-pocket costs were higher for all drug classes in Marketplace insurance than in Medicaid, with the largest adjusted monthly co-pay differences for the 3 categories consisting of only brand-name drugs (eTable 1 in the Supplement): $21.93 (95% CI, 19.25-24.60) for DPP-4 inhibitors, $50.03 (95% CI, 40.96-59.10) for GLP-1 agonists, and $20.72 (95% CI, 14.47-26.97) for SGLT-2 inhibitors (all *P* < .001). Total costs per month were also significantly higher in Marketplace insurance than in Medicaid for all drug classes except sulfonylureas, with adjusted differences ranging from $2.77 (95% CI, $1.70-$3.83) per prescription for metformin to $361.19 (95% CI, $251.90-$470.48) per prescription of GLP-1 agonists (eTable 1 in the Supplement). Out-of-pocket costs for non-insulin medications were 84.4% to 95.2% lower and total costs were 9.4% to 54.2% lower in Medicaid than in Marketplace plans. Out-of-pocket costs for insulin were 76.7% to 94.7% lower in Medicaid than in Marketplace plans, whereas differences in total insulin costs were mixed.

**Figure 2.**
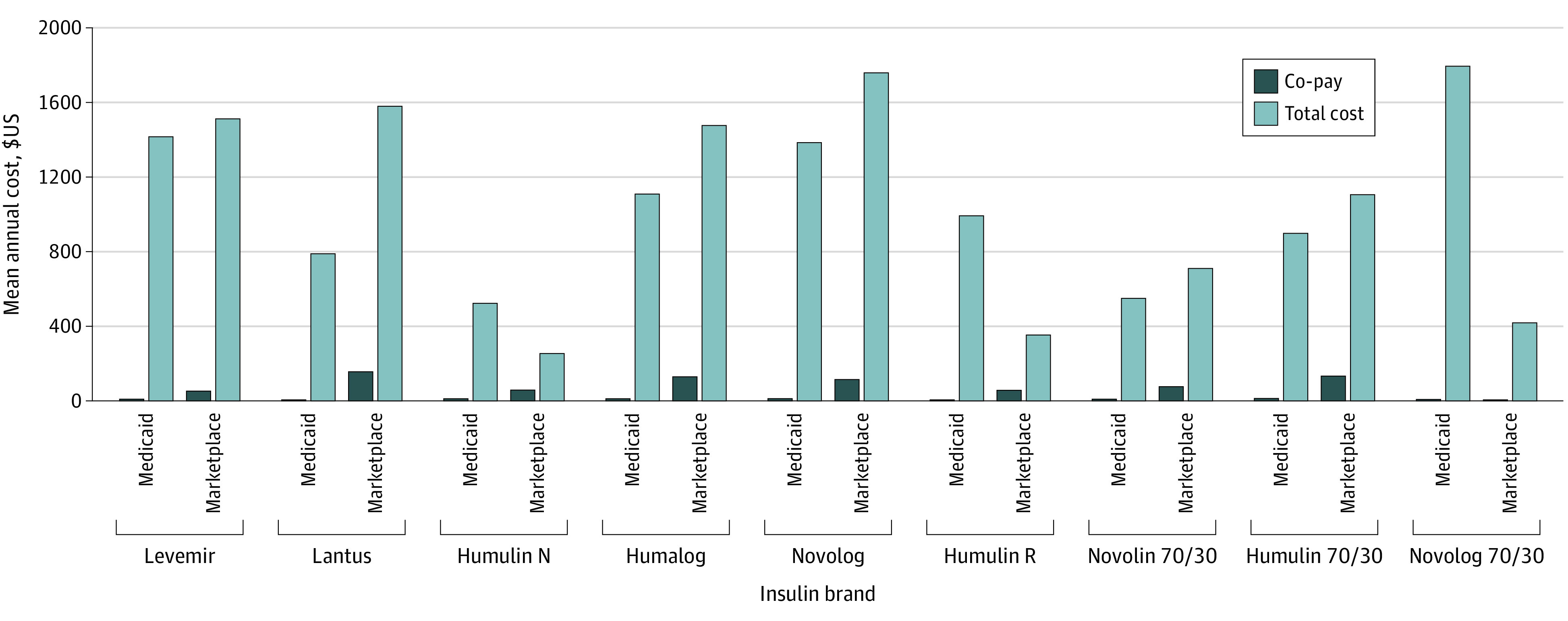
Annual Cost of Insulin Medications by Insurance Type The mean amounts presented exclude patients who were insured by both Medicaid and Marketplace plans in the same year. Humalog is insulin lispro; Humulin 70/30, 70% human insulin isophane suspension and 30% human insulin injection; Humulin N, insulin isophane; Humulin R, insulin human; Lantus, insulin glargine; Levemir, insulin detemir injection; Novolin 70/30, 70% human insulin isophane suspension and 30% regular human insulin injection; Novolog, insulin aspart; and Novolog 70/30, 70% insulin aspart protamine suspension and 30% insulin aspart injection.

Figure 2 shows the mean annual costs of the most commonly prescribed insulin brands by insurance type. Mean co-pays for the year were higher in Marketplace insurance than in Medicaid for all insulin brands. Total annual costs were generally higher in Marketplace insurance except for 3 insulin types. Total cost-sharing per prescription for most insulin brands was 4 to 15 times higher in Marketplace insurance than in Medicaid (eTable 2 in the Supplement). With regard to total costs per prescription, there were significantly higher costs in Medicaid for 1 insulin type, no significant differences for 5 insulin types, and significantly higher costs in Marketplace insurance for 3 insulin types (eTable 2 in the Supplement). When evaluating annual out-of-pocket costs for diabetic medications and for all pharmacy claims, we found that the mean total pharmacy out-of-pocket costs were $225.14 (95% CI, $213.69-$236.59; *P* < .001) higher in Marketplace plans compared with Medicaid in our adjusted model.

### Adherence to Noninsulin Antidiabetic Medications

Mean months covered with a prescription are presented in Table 3. Based on the months of active medication divided by months of coverage, adherence was similar for Marketplace insurance and Medicaid for all drugs except sulfonylureas, for which adherence was 5.3% (95% CI, 0.3%-10.4%; *P* = .04) higher in Marketplace plans than in Medicaid in the adjusted model. The percentage of patients who were undercovered or overprescribed antidiabetic medications in both Medicaid and Marketplace plans are shown in eTable 3 in the Supplement.

**Table 3.** Prescription Adherence for Noninsulin Antidiabetic Medications During 2014 and 2015^a^

Drug class	Months covered by prescription, No. (mean adherence, %)^b^		*P* value	Adjusted difference, % (95% CI)^e^	*P* value
	Medicaid-eligible patients^c^	Marketplace-eligible patients^d^			
DPP-4 inhibitors	8.45 (70.4)	8.75 (72.9)	.46	−1.3 (−9.7 to 7.0)	.76
GLP-1 agonists	7.97 (66.4)	7.56 (63.0)	.44	−10.7 (−22.8.1 to 1.4)	.08
SGLT-2 inhibitors	8.66 (72.2)	9.62 (80.2)	.15	6.4 (−8.7 to 21.6)	.41
Sulfonylureas	7.40 (61.7)	7.70 (64.2)	.20	5.3 (0.3 to 10.4)	.04
Metformin	7.26 (60.5)	7.30 (60.8)	.68	−0.1 (−2.5 to 2.2)	.92

Abbreviations: DPP-4, dipeptidyl peptidase 4; GLP-1, glucagon-like peptide 1; SGLT-2, sodium-glucose transport protein 2.

^a^ Data are from the Colorado All Payer Claims Database, linked to income data from Medicaid and Marketplace eligibility files, and are given at the member-year level.

^b^ Adherence was measured as months with an active prescription/months of coverage after receiving an active prescription.

^c^ Patients with income of 75% to 138% of the federal poverty level were eligible for Medicaid.

^d^ Patients with income of 139% to 200% of the federal poverty level were eligible for Marketplace insurance.

^e^ Model adjusted for age, sex, Elixhauser comorbidity index, income, and urban vs rural residence.

## Discussion

In this cross-sectional study of 22 788 diabetic adults with low income in Colorado, we found large savings in out-of-pocket costs for diabetes medications among those enrolled in Medicaid compared with those enrolled in Marketplace insurance plans; we also found differences in drug utilization patterns. The low out-of-pocket costs may reflect the better financial protection provided by Medicaid compared with subsidized private insurance (all of the Marketplace enrollees likely qualified for cost-sharing reductions under the ACA). We also found that patients with diabetes enrolled in Medicaid were significantly more likely to be taking newer medications such as DPP-4 inhibitors than were those enrolled in Marketplace insurance plans. Thus, although policy makers have voiced concerns about the scope of coverage in Medicaid compared with private insurance,^11^ in our study, this patient population with low income and at high risk of negative health outcomes appeared to have better access to newer (and more expensive) medications in Medicaid than in Marketplace plans and less financial burden in filling those prescriptions.

We found a similar trend among adult patients with low income taking insulin. The Medicaid group had lower out-of-pocket costs than the Marketplace group, with a difference of more than $30 per month per prescription in some cases. There were some differences in drug utilization across insulin types, but overall insulin utilization rates were similar. This finding likely reflects differences in insurance formularies by insulin type rather than a meaningful difference in access to insulin by insurance type.

Overall costs differed for most of the noninsulin medications, particularly for the 3 drug classes that were largely available as brand name only during the study period. This pattern likely reflects differences in the drug pricing rules for Medicaid vs private insurance plans. By statute, Medicaid programs receive the larger of a mandatory rebate and the best price that brand-name manufacturers offer to the private market as well as additional rebates to account for drug price increases over time that exceed inflation.^15,16^ Prices for brand-name oral noninsulin medications such as DPP-4 inhibitors and GLP-1 agonists have increased yearly since their introduction to the market starting in 2005, adding to the rebate that Medicaid programs receive. By contrast, brand-name manufacturers set drug prices for private markets at the level that they choose, and any negotiation that occurs with the Marketplace insurer or its pharmacy benefits manager results in smaller overall rebates. The overall cost differences of noninsulin medications between Medicaid and Marketplace plans has important insights for policy makers considering changes to the ACA or Medicaid policy that might impact adults with chronic conditions such as diabetes and quantifies some of the current cost savings associated with Medicaid drug policies.

When evaluating annual out-of-pocket costs for diabetic medications and for all pharmacy claims, we found that the mean total pharmacy out-of-pocket costs were $225.14 (95% CI, $213.69-$236.59; *P* < .001) higher in Marketplace plans compared with Medicaid in our adjusted model. The differential spending in Marketplace plans was disproportionately higher than the difference in income between Medicaid and Marketplace enrollees, further demonstrating that Medicaid provides cost savings for patients with diabetes.

We were able to conduct our analysis of drug adherence as a secondary outcome only for noninsulin medications owing to limitations in our data set on the quantity of insulin dispensed. We found that compared with patients with Medicaid, patients with Marketplace insurance had a greater percentage of months of apparent active medication coverage (our main proxy for medication adherence) for all drugs except GLP-1 agonists. Differences in adherence were statistically significant only for sulfonylureas, a result likely attributable to the small numbers of patients receiving brand-name–only oral diabetic medications in Marketplace plans, particularly SGLT-2 inhibitors.^17^

### Limitations

This study has limitations. The lack of information in the Colorado APCD on race and ethnicity precluded an assessment of whether these patterns of care differed across racial and ethnic subgroups. Also, because of the lack of clinical measures (such as indicators of glycemic control), we could not assess the overall adequacy of care being provided to patients in the sample. In addition, the claims database did not allow us to observe metal level, coupons and offsets, or formularies and preferred drug lists in Medicaid or Marketplace plans. Our data from 2014 and 2015 may not reflect current pricing or utilization patterns for diabetic medications, although the broad patterns by coverage type found in our study may provide a useful framework for future analyses using newer data. Similarly, our data set came from a single state, and specifics of plan formularies and negotiated prices may differ in other states.

Furthermore, our analysis used a cross-sectional design. Unmeasured confounders may explain some of the differences that we detected, although they are unlikely to explain the major cost differences in our results. Moreover, the novelty of the data linkages that we used enabled us to compare a relatively narrow band of adults with low income covered by the 2 types of insurance, which many previous comparisons of insurance type have been unable to do owing to crude or nonexistent measures of socioeconomic status in most claims data.

## Conclusions

Adults with low income in the US may qualify for Medicaid or subsidized Marketplace plans based on small differences in their income levels. In this cross-sectional study, we demonstrated that prescription drug costs for patients with diabetes were substantially lower for those enrolled in Medicaid than for comparable patients enrolled in subsidized Marketplace plans, suggesting that Medicaid drug pricing rules led to better control of pharmaceutical costs for these treatments than did Marketplace drug pricing rules. In addition, patients with diabetes had equal or better access to novel oral medications in Medicaid compared with subsidized Marketplace plans. Overall drug costs were substantially lower for noninsulin medications in Medicaid than in Marketplace insurance, whereas differences in insulin costs were variable. In our study, we also found that patients with diabetes experienced broader access to expensive prescription drugs in Medicaid than in private plans.

## Notes

Supplement. **eFigure.** Sample Construction **eTable 1.** Costs of Most Used Non-Insulin Antidiabetics, $USD, 2014-2015 **eTable 2.** Costs of Most Used Brands of Insulin, $USD, 2014-2015 **eTable 3.** Undercovered and Overprescribed, Non-Insulin Antidiabetic Medications, 2014-2015 (Member-Year Level)
