# Supplementary material for: Differences in Diabetic Prescription Drug Utilization and Costs Among Patients With Diabetes Enrolled in Colorado Marketplace and Medicaid Plans, 2014-2015
Source: JAMA Netw Open. 2022 Jan 14;5(1):e2140371. doi: 10.1001/jamanetworkopen.2021.40371 (PMC8760612; doi:10.1001/jamanetworkopen.2021.40371)
Supplement: Supplement. — eFigure. Sample Construction eTable 1. Costs of Most Used Non-Insulin Antidiabetics, $USD, 2014-2015 eTable 2. Costs of Most Used Brands of Insulin, $USD, 2014-2015 eTable 3. Undercovered and Overprescribed, Non-Insulin Antidiabetic Medications, 2014-2015 (Member-Year Level) [file jamanetwopen-e2140371-s001.pdf]

Supplemental Online Content

Khorrami P, Sinha MS, Bhanja A, Allen HL, Kesselheim AS, Sommers BD. Differences in diabetic prescription drug utilization and costs among patients with diabetes enrolled in Colorado Marketplace and Medicaid plans, 2014-2015. *JAMA Netw Open.* 2022;5(1):e2140371. doi:10.1001/jamanetworkopen.2021.40371

eFigure. Sample Construction

eTable 1. Costs of Most Used Non-Insulin Antidiabetics, $USD, 2014-2015

eTable 2. Costs of Most Used Brands of Insulin, $USD, 2014-2015

eTable 3. Undercovered and Overprescribed, Non-Insulin Antidiabetic Medications, 2014-2015 (Member-Year Level)

© 2022 Khorrami P et al. *JAMA Network Open.*

eFigure. Sample Construction

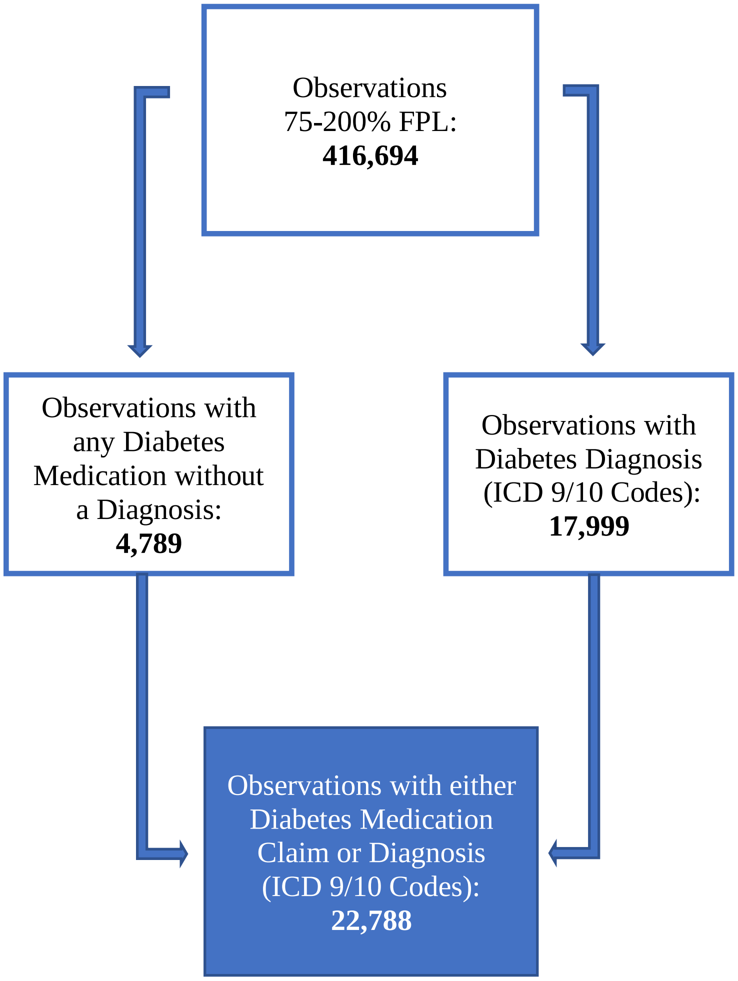

Source: Authors analysis of Colorado all payer claims database from 2014-2015
Notes: Observations were counted at the member-year level, the counts presented are 2014-2015 combined totals.

© 2022 Khorrami P et al. *JAMA Network Open.*

eTable 1. Costs of Most Used Non-Insulin Antidiabetics, $USD, 2014-2015

	Unadjusted			Adjusted		
**Drug Name**	**Medicaid Eligible (75-138% FPL)**	**Marketplace Eligible (139-200% FPL)**	**P value**	**Adjusted Difference**	**95% Confidence Interval**	**P value**
Panel A: Average Copay Per Month						
DPP-4 Inhibitors	1.42	25.41	<0.001	21.93	(19.25, 24.60)	<0.001
GLP-1 Agonists	2.51	51.90	<0.001	50.03	(40.96, 59.10)	<0.001
SGLT-2 Inhibitors	1.70	27.10	<0.001	20.72	(14.47, 26.97)	<0.001
Sulfonylureas	0.65	4.16	<0.001	3.36	(3.12, 3.61)	<0.001
Metformin	0.70	4.71	<0.001	3.72	(3.57, 3.86)	<0.001
Panel B: Average Total Cost Per Month						
DPP-4 Inhibitors	161.78	234.66	<0.001	95.07	(59.15, 130.99)	<0.001
GLP-1 Agonists	301.62	618.14	<0.001	361.19	(251.90, 470.48)	<0.001
SGLT-2 Inhibitors	135.57	295.88	<0.001	147.54	(87.48, 207.60)	<0.001
Sulfonylureas	10.73	11.84	0.16	1.11	(-1.86, 4.07)	0.47
Metformin	7.12	9.35	<0.001	2.77	(1.70, 3.83)	<0.001

Notes:
Data are from the Colorado All Payer Claims Database, linked to income data from Medicaid and Marketplace eligibility files. All cost estimates were mean costs in 2015 inflation-adjusted terms.
Models adjusted for age, sex, Elixhauser comorbidity index, income, and urban vs. rural residence.

© 2022 Khorrami P et al. *JAMA Network Open.*

eTable 2. Costs of Most Used Brands of Insulin, $USD, 2014-2015

		Unadjusted			Adjusted		
**Insulin Group**	**Drug Name**	**Medicaid Eligible (75-138% FPL)**	**Marketplace Eligible (139-200% FPL)**	**P value**	**Adjusted Difference**	**95% Confidence Interval**	**P value**
Panel A: Average Copay Per Prescription							
Basal / Background	Levemir	2.64	12.94	<0.001	9.86	(8.40, 11.31)	<0.001
	Lantus	1.86	34.85	<0.001	30.95	(27.63, 34.27)	<0.001
	Humulin N	3.62	17.48	<0.001	11.47	(7.79, 15.15)	<0.001
Bolus / Mealtime	Humalog	2.44	35.82	<0.001	31.70	(27.69, 35.71)	<0.001
	Novolog	3.34	27.14	<0.001	23.33	(18.62, 28.05)	<0.001
	Humulin R	2.93	19.21	<0.001	16.92	(11.39, 22.44)	<0.001
Pre-mixed / Combination	Novolin 70/30	3.57	24.94	<0.001	16.62	(7.49, 25.75)	<0.001
	Humulin 70/30	2.30	9.88	0.04	6.67	(4.26, 9.07)	<0.001
	Novolog 70/30	2.33	1.68	0.38	-0.64	(-1.75, 0.48)	0.26
Panel B: Average Total Cost Per Prescription							
Basal / Background	Levemir	361.41	405.30	0.20	37.47	(-84.89, 159.83)	0.55
	Lantus	140.84	359.52	<0.001	227.1	(184.88, 269.35)	<0.001
	Humulin N	138.64	70.38	<0.001	-63.68	(-100.49, -26.86)	0.001
Bolus / Mealtime	Humalog	232.76	350.25	<0.001	160.06	(101.88, 218.24)	<0.001
	Novolog	440.34	518.37	0.04	112.47	(17.76, 207.18)	0.02
	Humulin R	219.23	145.65	0.07	-9.88	(-158.49, 138.73)	0.90
Pre-mixed / Combination	Novolin 70/30	213.90	157.16	0.14	-86.78	(-216.48, 42.91)	0.19

© 2022 Khorrami P et al. *JAMA Network Open.*

	Humulin 70/30	119.17	135.65	0.69	-28.18	(-146.53, 90.17)	0.64
	Novolog 70/30	444.58	265.34	0.18	32.96	(-439.28, 505.20)	0.89

Notes:
Data are from the Colorado All Payer Claims Database, linked to income data from Medicaid and Marketplace eligibility files.
All cost estimates were mean costs in 2015 inflation-adjusted terms.
Models adjusted for age, sex, Elixhauser comorbidity index, income, and urban vs. rural residence.

© 2022 Khorrami P et al. *JAMA Network Open.*

eTable 3. Undercovered and Overprescribed, Non-Insulin Antidiabetic Medications, 2014-2015 (Member-Year Level)

	Undercovered			Overprescribed		
**Drug Class**	**Medicaid Eligible (75-138% FPL)**	**Marketplace Eligible (139-200% FPL)**	**P value**	**Medicaid Eligible (75-138% FPL)**	**Marketplace Eligible (139-200% FPL)**	**P value**
DPP-4 Inhibitors	0.19%	0%	0.08	38.4%	34.4%	0.43
GLP-1 Agonists	0.63%	0%	0.05	31.4%	15.3%	0.002
SGLT-2 Inhibitors	0.62%	0%	0.16	37.8%	32.4%	0.53
Sulfonylureas	0.34%	0.67%	0.49	22.1%	16.6%	0.02
Metformin	0.21%	0.54%	0.08	20.1%	11.3%	<0.001

Notes:
Data are from the Colorado All Payer Claims Database, linked to income data from Medicaid and Marketplace eligibility files.

© 2022 Khorrami P et al. *JAMA Network Open.*
